# Mortality in patients with non-functioning pituitary adenoma

**DOI:** 10.1007/s11102-018-0863-9

**Published:** 2018-01-17

**Authors:** Metaxia Tampourlou, Athanasios Fountas, Georgia Ntali, Niki Karavitaki

**Affiliations:** 1 Institute of Metabolism and Systems Research, College of Medical and Dental Sciences, University of Birmingham, IBR Tower, Level 2, Birmingham, B15 2TT UK; 2 Centre for Endocrinology, Diabetes and Metabolism, Birmingham Health Partners, Birmingham, B15 2TH UK; 3 Department of Endocrinology, Queen Elizabeth Hospital, University Hospitals Birmingham NHS Foundation Trust, Birmingham, B15 2TH UK; 4 Department of Endocrinology, Diabetes and Metabolism, Evangelismos Hospital, 10676 Athens, Greece

**Keywords:** Non-functioning pituitary adenomas, Mortality, Hypopituitarism

## Abstract

Non-functioning pituitary adenomas (NFA) are benign pituitary neoplasms not associated with clinical evidence of hormonal hypersecretion. A substantial number of patients with NFA have morbidities related to the tumor and possible recurrence(s), as well as to the treatments offered. Studies assessing the long-term mortality of patients with NFA are limited. Based on the published literature of the last two decades, overall, the standardized mortality ratios in this group suggest mortality higher than that of the general population with deaths attributed mainly to circulatory, respiratory and infectious causes. Women seem to have higher mortality ratios, and assessment of time trends suggests improvement over the years. There is no consensus on predictive factors of mortality but those most consistently identified are older age at diagnosis and high doses of glucocorticoid substitution therapy. Well designed and of adequate power studies are needed to establish the significance of factors compromising the survival of patients with NFA and to facilitate improvements in long-term prognosis.

## Introduction

Non-functioning pituitary adenomas (NFA) or, as recently proposed, non-functioning pituitary neuroendocrine tumors [1] are benign pituitary neoplasms that arise from the adenohypophyseal cells and are not associated with clinical evidence of hormonal hypersecretion. They constitute 15–37% of all pituitary adenomas [2–4] and have a prevalence of 70–220 per million inhabitants [2–5]. The absence of clinical manifestations of hormonal hypersecretion relates with diagnostic delay and, with the exception of incidentally found ones, they are usually detected when they are large enough to cause pressure effects to surrounding structures. When treatment is indicated, surgery is the first option, combined or not with adjuvant radiotherapy [6].

A substantial number of patients with NFA have morbidities related to the tumor and possible recurrence(s), as well as to the treatments offered; visual deterioration and hypopituitarism are the most frequent [6–10]. As a result of these, the long-term survival of these patients may be compromised.

In this review, we have evaluated and summarised the literature published in the last two decades on the mortality of patients with NFA and potential factors affecting it. Series also involving subjects with other (para)sellar tumors and not analysing the outcomes of those with NFA separately were not included.

### Mortality in patients with non-functioning pituitary adenomas

Studies assessing the long-term mortality of patients with NFA are limited and, overall, they suggest mortality higher than that of the general population. A summary of the literature reporting standardized mortality ratios (SMR) that are included in this review is shown in Table 1.

**Table 1:** Studies reporting standardized mortality ratios in patients with non-functioning pituitary adenoma (those reviewing a subgroup of a previously studied population are not included)

Study	Country period covered	Number of patients	Surgery/radiotherapy	Follow-up (years) (range)	Standardized mortality ratio (95% CI)
Ntali et al. [8]	UK 1963–2011	546	100%/29%	Median 8.0 (1 month–48.5)	3.6 (2.9–4.5)
Nielsen et al. [11]	Denmark 1985–1996	192	100%/22%	Median 13.1 (8.0–20.0) (surviving subjects) 4.6 (0–18.6) (deceased subjects)	1.21 (0.93–1.59)
Tomlinson et al. [14]	UK 1992–2000	573 (all had hypopituitarism)	90%/42%	Not reported for patients with NFA	1.70 (1.34–2.15)
Olsson et al. [15]	Sweden 1987–2011	2795	Patients diagnosed before 1997 (12%) Treatment not available Patients diagnosed after 1997 (88%) 52%/4%	Mean 7 (0–25)	1.10 (1.00-1.20)

Nielsen et al. [11], in a series of 192 patients from three Danish centers treated surgically between 1985 and 1996 (in 83% by transsphenoidal approach − 22% had received adjuvant radiotherapy), found SMR of 1.21 (95% CI, 0.93–1.59); the median follow-up for the surviving subjects was 13.1 years and for those who died 4.6 years. SMR was significantly increased in women (2.11; 95% CI, 1.35–3.31) but not in men and the causes of death were not cardiovascular (further details are not provided). Mortality was also reported similar in patients who had been offered radiotherapy and in those having only surgery. The relatively small number of patients remains a limitation of this report. In a further study from the same centers covering the same period and including 160 patients [12], SMRs of patients with normal pituitary function, partial hypopituitarism or panhypopituitarism were not different from the general population; it should be noted, however, that the management of pituitary hormone deficits is not clarified in this paper.

Chang et al. [13], in a series of 663 patients with NFA managed between 1975 and 1995 (transsphenoidal approach in most of the cases − 51% had received adjuvant radiotherapy) in US centers and followed up for a median time of 14 years, reported that the study population had a significantly higher mortality rate than the general population; however, no SMRs or causes of death were provided.

Tomlinson et al. [14], in a series of 573 patients with NFA included in the West Midlands Hypopituitary database (42% of them also had radiotherapy), found SMR of 1.70 (99% CI, 1.34–2.15) with the excess mortality explained by an increase in respiratory and cardio/cerebrovascular deaths. Mortality was increased in women comparatively with men (SMR 2.23; 99% CI, 1.60–3.11 vs. SMR 1.37; 99% CI, 0.98–1.91, *p* = 0.006). It should be pointed out that this study may have been affected by selection bias, as it includes only subjects with NFA and hypopituitarism. Furthermore, almost one-third of the patients with gonadotrophin deficiency did not receive replacement therapy and adrenocorticotropic hormone (ACTH) deficiency was treated with higher doses than currently used (15–30 mg hydrocortisone per day). Finally, around 10% of the patients had no surgery and, therefore, no histological confirmation of the adenoma diagnosis.

Olsson et al. [15], in a series of 2795 patients identified in the Swedish National Patient Registry and followed up for a mean period of 7 years, reported a SMR of 1.10 (95% CI, 1.00–1.20). Increased mortality was described in patients diagnosed before the age of 40 years (SMR 2.68; 95% CI, 1.23–5.09), in women (SMR 1.29; 95% CI, 1.11–1.48) and in those treated with radiotherapy (SMR 2.67; 95% CI, 1.79–3.84). In contrast to males, females with hypopituitarism or diabetes insipidus had increased SMR (1.38; 95% CI, 1.12–1.66 and 2.43; 95% CI, 1.26–4.25, respectively). Deaths were attributed to infectious and circulatory diseases. A significant advantage of this study is its large sample size but a drawback is the validity of the diagnosis in the reviewed cases (when the identification criteria were checked in a group of 467 subjects, the presence of NFA was confirmed in 90.8% of them; 1172 patients diagnosed between 1997 and 2011 had no surgery or radiotherapy, thereby lacking histological confirmation and information on possible surgical treatments and radiotherapy were not available for those diagnosed before 1997). Furthermore, hypopituitarism was defined by an entry of this term in the Registry and details on number of patients checked, diagnostic criteria, degree and management of hypopituitarism were not available. Assessment of time trends in mortality in a subgroup of patients from the same Registry revealed that in females, this was increased for those diagnosed between 1997 and 2006 but not between 2007 and 2011 [16]. The authors reported that during the study period, the prevalence of diabetes insipidus and the use of surgery and radiotherapy had remained stable but the frequency of hypopituitarism had declined. Nonetheless, as in their previous study [15], details on the diagnosis and management of the pituitary hormone deficits were not available affecting the interpretation of the results.

Ntali et al. [8], in a series of 546 patients operated on for a macroNFA between 1963 and 2011 and followed up for a median period of 8 years in a UK center, reported SMR of 3.6 (95% CI, 2.9–4.5). Transsphenoidal surgery had been offered to 97% of the patients and 29% of them had received adjuvant radiotherapy. SMR was 4.7 (95% CI, 2.7–7.6) for those operated before 1990 and 3.5 (95% CI, 2.8–4.4) for those operated after 1990. Main causes of death were due to cardio/cerebrovascular diseases (34%), infections (30%) and malignancies (29%). In a subset of patients with adequate clinical follow-up data (n = 436), the SMR was 3.8 (95% CI, 2.0–4.7); in this group, 97% of the patients had transsphenoidal surgery, 25.3% had regrowth of the NFA and 43.1% had received radiotherapy (adjuvant post-operative or for relapse).

Based on these data which mostly rely on European populations, overall, the SMR of patients with NFA are increased with deaths attributed mainly to circulatory, respiratory and infectious causes. Women seem to have higher mortality ratios and assessment of time trends suggests improvement over the years.

### Predictive factors of mortality

Cox regression analyses have been performed in a number of studies aiming to identify predictive factors of mortality. A summary of the published data is discussed below. The confounding factors used in Cox model were often different between the studies and this may partially explain the variability of the results.

### Age at diagnosis

Ntali et al. [8], have shown that age at diagnosis was an independent predictor of mortality [after adjustment for factors proven to be significant in univariate analysis in this series: radiotherapy, regrowth of tumor and untreated growth hormone (GH) deficiency] with shorter survival for older patients [Hazard ratio (HR) 1.10; 95% CI, 1.07–1.13, *p* < 0.001]. In this study, a significant difference in the rate of survival between subjects diagnosed before and after the age of 50 years was also noted (survival at 10 years 98.5% vs. 80.9% respectively, *p* < 0.01). Similar results were described by Chang et al. [13] with HR 1.09 (95% CI, 1.08–1.84, *p* < 0.0001). On the other hand, Olsson et al. [15] reported that young age at diagnosis was a strong predictor of mortality with HR for those diagnosed at the age of 40 or earlier of 3.47 (95% CI, 1.28–9.42, *p* = 0.015) for males and 4.20 (95% CI, 1.32–13.32, *p* = 0.015) for females. The explanation for this observation remains unclear.

### Sex

Despite the increased SMRs reported in women, sex has not been confirmed as an independent predictor of mortality [8, 13, 17]. The adjustment for possible confounding factors is a potential explanation for this discrepancy.

### Radiotherapy

In most recent series of patients with NFA, radiotherapy has not shown to be an independent predictor of mortality [8, 13, 18]. On the other hand, Olsson et al. [15] reported that radiotherapy was a predictive factor of excess mortality (males HR 1.99; 95% CI, 1.15–3.42, *p* = 0.014; females HR 2.81; 95% CI, 1.63–4.83, *p* < 0.0001). It should be mentioned, however, that only 4% of their patients had received irradiation. Notably, in a subsequent study including a subgroup of patients diagnosed with NFA between 1997 and 2011 and with the end of the study extended for three further years, Olsson et al. [19] found that radiotherapy did not have an effect on the Cox model for mortality. Van Varsseveld et al. [20], in a series of 806 patients with NFA and severe GH deficiency identified from the Dutch National Registry of Growth Hormone Treatment in Adults (96% of them were treated with GH and 57% had received radiotherapy) and followed up for a median period of 10 years, found that after adjustment for relevant confounders (age, gender, treated ACTH insufficiency and GH replacement), mortality was not different between irradiated and non-irradiated patients. The impact of other pituitary hormone deficits and their management was not taken into account in this study.

### Pituitary hormone deficiencies

The clarification of the impact of hypopituitarism on the mortality of patients with NFA based on the published literature is confounded by a number of factors including the variable criteria applied for the diagnosis of pituitary hormone deficits (especially for studies covering long periods), the differences in the hormone substitution protocols between centers and between studies and the developments/improvements in the management of hypopituitarism over the years [21].

- GH deficiency: Ntali et al. [8] found that untreated GH deficiency was not an independent predictor of mortality. In this series, selection bias may have affected the reliability of the results, as in the UK, GH replacement is offered after prescreening of patients according to the National Institute of Clinical Excellence (NICE) guidelines and those with an optimal score in the Quality of Life assessment standardized questionnaire (Assessment of Growth Hormone Deficiency in Adults—AGHDA) are not candidates for treatment. O’Reilly et al. [18] reported that GH deficient patients were not at increased risk of death compared to those with intact GH secretion after adjusting for surgery, age at diagnosis, attained age, sex and radiotherapy. However, when considered separately, patients with untreated GH deficiency had higher mortality risk compared to those with treated GH deficiency; the reason for this discrepancy is not clear. Olsson et al. [19] found that after adjusting for starting age in the study, patients treated with GH had decreased risk for mortality in comparison with non-GH treated (HR 0.94; 95% CI, 0.91–0.98, *p* = 0.0063); the latter group also included GH replete patients and other factors like radiotherapy, body mass index, gender, different replacement therapies (on which further details were not provided) did not have a significant effect on the Cox model and were not selected for the final statistical model.
- Gonadotrophin deficiency: O’Reilly et al. [18] found that the mortality risk was higher in gonadotrophin deficient compared with gonadotrophin preserved patients after correction for factors reported above (Relative Risk 2.56; 95% CI 1.10–5.96, *p* = 0.01); following sub-analysis of gonadotrophin deficiency according to sex, and after additional adjustment for dysfunction of all other pituitary hormone axes, significant association with increased mortality remained only in men (Relative Risk 5.44; 95% CI, 1.07–27.69, *p* = 0.02). Interestingly, in this series, sex hormone replacement therapy was prescribed only for 79.8 and 21.5% of male and female gonadotrophin deficient patients, respectively. Ntali et al. [8] found that untreated gonadotrophin deficiency was not an independent predictor of survival, but in this study a very small percentage of deficient subjects had remained without sex hormone substitution.
- ACTH deficiency: Zueger et al. [22], in a series of patients with NFA and ACTH deficiency concluded that the HR was 1 for patients on a total dose of hydrocortisone between 5 and 19 mg daily but increased significantly to 4 when the total dose was higher than 30 mg daily. In agreement with these findings, O’Reilly et al. [18] after multivariate analysis, found that the relative risk of death was increased in patients offered total daily dose of hydrocortisone higher than 30 mg (Relative Risk 3.79; 95% CI, 1.49–9.67, *p* < 0.01). These observations are also supported by Ntali et al. [8] who did not identify ACTH deficiency as a predictor of mortality; in this series, around 90% of the patients were on a lower dose, 20 mg total daily dose of hydrocortisone. Finally, Hammarstrand et al. [17] after Cox-regression analysis adjusted for age at the start of study, gender and radiotherapy, showed that patients receiving a daily hydrocortisone equivalent dose of more than 20 mg had significantly increased mortality compared to subjects not on glucocorticoid replacement (HR 1.88; 95% CI, 1.06–3.33, *p* = 0.032).
- Thyroid-stimulating hormone (TSH) deficiency: In the study by O’Reilly et al. [18], the relative risk of death was increased in patients on levothyroxine daily dose less than 100 mcg. This finding needs to be interpreted cautiously as the levels of peripheral thyroid hormones achieved by each dose had not been taken into account. On the other hand, Ntali et al. [8] did not identify TSH deficiency as a predictor of mortality; in this series, levothyroxine replacement was offered in a dose aiming to produce a free thyroxine level within the reference range (preferably in the upper half).
- Vasopressin deficiency: Treatment with desmopressin or diabetes insipidus have not been shown to be predictors of mortality in patients with NFA [8, 15, 18].

### Other factors

Regrowth of the NFA, presentation with acute apoplexy, cavernous sinus invasion, extent of adenoma removal and repeat surgery for relapse were not predictors of mortality in a recent study [8]. Furthermore, larger tumor size (> 25 mm) and extent of NFA resection were not independent predictors of mortality either [13].

## Conclusions and future perspectives

Based on the most recent literature, mortality in patients with NFA is increased with the survival being more compromised possibly in females and in patients diagnosed at an older age. The advances in the management of these patients (and particularly in the substitution of pituitary hormone deficits) has had a positive impact on survival. Nonetheless, further well designed and of adequate power studies are needed to establish the significance of other factors preventing the achievement of mortality similar to that of the general population. Series from non-European centers would also be valuable for this aim and would further expand the field.
